# Mutational insights into human kynurenine aminotransferase 1: modulation of transamination and β-elimination activities across diverse substrates

**DOI:** 10.1042/BCJ20253178

**Published:** 2025-08-18

**Authors:** Arun Kumar Selvam, Renhua Sun, Ali Razaghi, Hugh Salter, Tatiana Sandalova, Mikael Björnstedt, Adnane Achour

**Affiliations:** 1 Division of Pathology, Department of Laboratory Medicine, Karolinska Institutet, Stockholm, SE-141 86, Sweden; 2 Science for Life Laboratory, Department of Medicine Solna, Karolinska Institute, & Division of Infectious Diseases, Karolinska University Hospital, SE-171 77, Solna, Sweden

**Keywords:** amino acid metabolism, aminotransferase, β-elimination, human kynurenine aminotransferase, Se-methylselenocysteine, tryptophan metabolism

## Abstract

Human kynurenine aminotransferase 1 (hKYAT1) plays a crucial role in the transamination of aromatic amino acids and kynurenine. This promiscuous homodimeric enzyme transaminates various amino acids into their corresponding α-keto acids. Additionally, hKYAT1 is known to catalyze the β-elimination of cysteine-S conjugates and cysteine-Se conjugates. In this study, we performed mutational analyses of hKYAT1, targeting its catalytic, ligand-binding, and substrate-binding sites. The transamination activity of 13 mutant variants was systematically evaluated against sixteen different amino acid substrates, including kynurenine, selenomethionine (SeMet), and Se-methylselenocysteine (MSC), as well as for the β-elimination of SeMet and MSC. Our results demonstrate that mutations of residues E27 in the catalytic site and H279 in the substratestabilizing site significantly enhanced the transamination of several amino acids, including phenylalanine, tryptophan, histidine, and MSC. The H279F mutation increased transamination and β-elimination of MSC by 2- and 1.5-fold, respectively. Furthermore, mutation at the ligand-binding residues R398, F125, and N185 substantially reduced MSC transamination activity of hKYAT1. Interestingly, none of the tested mutations affected the transamination of l-kynurenine, a natural substrate of hKYAT1. Altogether, these findings support future investigation into hKYAT1 as a modifiable target in selenium-mediated anticancer approaches.

## Introduction

The metabolism of amino acids is essential for the proliferation and maintenance of all living cells. Several amino acids serve as neurotransmitters, regulate the cellular redox status, and are involved in critical pathways including the activation of the mTOR pathway or DNA methylation [1,2]. Notably, rapidly proliferating cells, i.e., immune or tumor cells, exhibit a greater demand for amino acids compared with normal cells [1,2]. Therefore, the anabolic and catabolic regulation of amino acids is essential for normal cellular function and constitutes a critical pathway in numerous diseases.

Transamination is a pivotal step in the catabolism of a large ensemble of amino acids. To date, at least six distinct human cytosolic transaminases have been identified, all involved in amino acid catabolic pathways [3]. Notably, cytosolic kynurenine aminotransferase (KYAT1) catalyzes the conversion of kynurenine (Kyn) to kynurenic acid (Kyna), a key reaction within the kynurenine pathway (KP), the primary catabolic route for tryptophan. The KP comprises a complex cascade of reactions catalyzed by eight different enzymes [4]. Tryptophan metabolism occurs in most cells and plays important roles in various organs, including the kidneys, liver, heart, lungs, and cells in the brain such as astrocytes and microglia [5].

Human kynurenine aminotransferase 1 (hKYAT1, also known as KAT1) is part of the type I aminotransferase subfamily, with aspartate aminotransferase as its prototypical member [6]. The activity of aspartate aminotransferases is dependent on the cofactor pyridoxal 5′-phosphate (PLP), which enables the transfer of the amine group from the substrate amino acid to the formed α-keto acid [7]. To date, four human KYAT variants (hKYAT1–hKYAT4) have been identified [4], with broad substrate specificity. Additionally, hKYAT1 and hKYAT3 exhibit β-lyase activity toward cysteine-S conjugate and cysteine-Se conjugate substrates that possess strong electron-withdrawing groups at the sulfur or selenium atom [8]. Notably, *in vitro* studies have demonstrated that hKYAT1 exhibits broad substrate specificity [8], achieving its highest catalytic efficiency with l-glutamine (l-Gln), l-phenylalanine (l-Phe), l-leucine (l-Leu), l-tryptophan (l-Trp), l-kynurenine (l-Kyn), and l-methionine (l-Met) [9]. Additionally, it also exhibits significant β-lyase activity with S-(1,2-dichlorovinyl)-l-cysteine (DCVC), S-(1,1,2,2-tetrafluoroethyl)-l-cysteine (TFEC), and MSC [10]. This broad functional capacity accounts for the various names attributed to hKYAT1, such as kynurenine aminotransferase 1 (EC 2.6.1.7), glutamine transaminase K (GTK) (EC 2.6.1.64), and cysteine conjugate β-lyase 1 (EC 4.4.1.13).

hKYAT1 has been implicated in various diseases, including cancer [11], neurodegenerative and psychiatric disorders [12], inflammation, and obesity [13], primarily due to its role in tryptophan metabolism within the KP. Altered hKYAT1 activity within the KP has been associated with conditions such as cerebral ischemia [14], while significantly reduced hKYAT1 mRNA expression has been linked to severe skeletal disorders [15]. Notably, large amounts of compounds produced along the KP play important roles in modulating different biological processes within the organism [16–19]. However, the significance of hKYAT1 extends beyond the KP, as it is a key component of the glutaminase II pathway. This enzyme catalyzes the transamination of glutamine to α-ketoglutaramate (KGM) in the presence of an α-keto acid acceptor. This reaction is coupled to α-amidase, which hydrolyzes KGM to α-ketoglutarate, facilitating its entry into the tricarboxylic acid (TCA) cycle [10]. Many cancers rely on increased glutamine catabolism for energy production, often due to the decoupling of the glycolytic pathway from the TCA cycle [20]. Interestingly, both human KYAT1 and KYAT3 have been found to be overexpressed in various cancer cell lines [10,21]. Several crystal structures of the hKYAT1 holoenzyme and hKYAT1 in complex with an array of substrates or inhibitors have been previously determined [22–24]. While more than 500 different point mutations have been identified in hKYAT1, none have been produced or functionally characterized. To date, no mutational studies have clarified the role of critical residues in hKYAT1. In this study, we present the first comprehensive mutational analysis of the substrate-binding region of hKYAT1. By evaluating the impact of various mutations on both transamination and α-elimination activities using a diverse range of substrates, we aimed to elucidate the molecular determinants involved in substrate recognition by hKYAT1. These findings provide a foundation for the rational design of selective inhibitors with potential therapeutic applications.

## Results

### Structure-based selection and design of mutations

**Figure 1:**
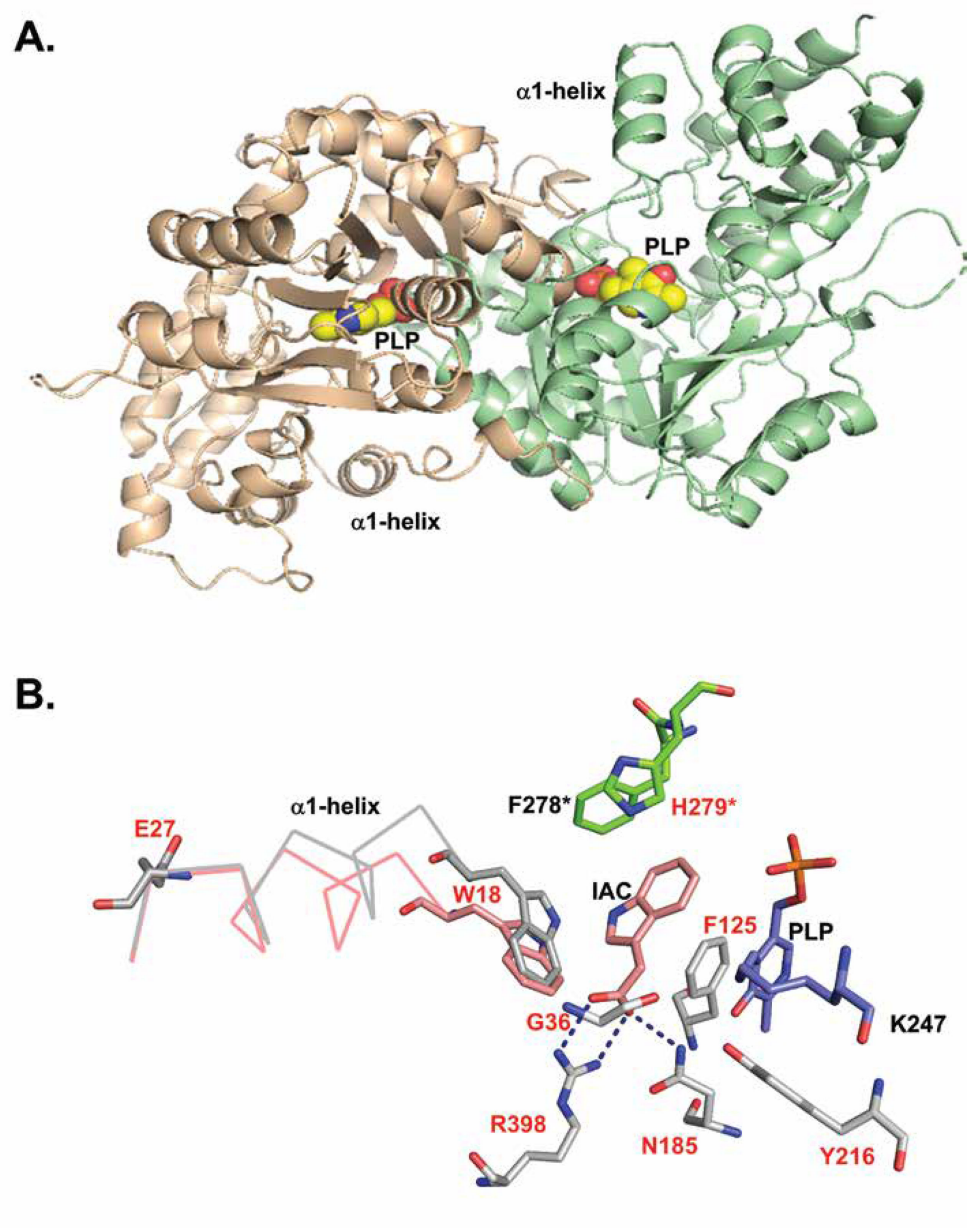
Structural rationale for active site residue selection in hKYAT1. (**A**) Ribbon diagram of hKYAT1 homodimer showing subunits in pink and light green. The cofactor PLP marks the active sites. The N-terminal α1-helix (residues 18–27), crucial for catalytic activity, is highlighted. (**B**) Overlay of ligand-free (gray) and IAC-bound (pink) (PDB code: 3FVU) [22] hKYAT1 structures reveals lateral displacement of the α1-helix upon ligand binding. Active site residues from each subunit are shown (gray and green). Mutated residues in this study are labeled in red. hKYAT1, human kynurenine aminotransferase 1; PLP, pyridoxal 5´-phosphate.

Several crystal structures of hKYAT1 have been resolved in complex with the co-factors PLP (PDB codes 1W7L [24], 3FVS [22], and 4WLH [23]), or pyridoxamine 5′-phosphate (PMP, 1W7N [24]), as well as substrates like l-Phe (1W7M [24]), and inhibitors including indole-3-acetic acid (3FVU [22]). hKYAT1 forms a stable homodimer with a buried interface surface area of approximately 3000 Å^2^ (Figure 1A). The substrate binds above the PLP ring, which is covalently linked to the lysine residue K247 in the active site of hKYAT1. This pocket is composed of residues from both subunits (Figure 1B). To investigate how structural features influence enzyme function, we performed targeted mutagenesis to alter substrate-interacting residues while preserving PLP-binding integrity. Our goal was to evaluate how specific side-chain substitutions affect substrate recognition and catalytic activity.

The crystal structure of hKYAT1 complexed with the substrate l-Phe [24] or the inhibitor IAC [22] reveals the substrate positioned between residues F125 and H279* (Figure 1B, * indicates residues from the other subunit). Residues N185 and R398 form hydrogen bonds and salt bridges with the substrate’s α-carboxylate group, positioning its α-amino group above the PLP in a conformation conducive to catalysis. The ligand-binding site of hKYAT1 is composed of several aromatic residues, including W18, Y101, and F125 from one monomer, and Y63*, H279*, and F278* from the other subunit. To investigate the role of these aromatic residues, we introduced mutations at key positions. F125H and H279F were predicted to modulate—but not abolish—substrate binding, potentially altering activity toward aromatic and non-aromatic substrates. R398A was introduced to test whether N185 alone could anchor the substrate. A double mutant, N185G/R398K, was designed to determine whether lysine at position 398 could compensate for the loss of N185. In addition, N185 was mutated to glutamine (N185Q) to reduce side-chain volume while maintaining polar character. Based on prior evidence that tyrosine residues can substitute for arginine in substrate anchoring [22], we generated individual and combined Y216R and R398A mutants. G36S mutant was designed to narrow the binding pocket, potentially improving substrate selectivity.

Unlike subgroup I aminotransferases, such as aspartate aminotransferase, which undergo conformational changes upon ligand binding to close the active site [7], hKYAT1 binds substrates without significant domain movement. Instead, substrate-binding involves a shift in the N-terminal α1-helix (residues P17–E27) with a pivotal movement facilitated by the glutamate residue E27. This shift tilts the α1-helix toward the active site and is accompanied by a rotation of Y101 (Figure 1B). The proximity between W18 and the substrate is reduced to 3.9 Å, creating optimal van der Waals interactions (Figure 1B). To test the role of this helix shift, we mutated E27 to glycine (E27G) to increase helix flexibility and substituted W18 with smaller residues (H, M, L) to evaluate the impact of side-chain on substrate specificity (Figure 1B).

All designed mutations are listed in Supplementary Table S1. Most activity assays were performed using cell lysates; however, to minimize interference from endogenous factors, five key mutants (H279F, F125H, N185Q, N185G/R398K, and R398A) were purified and tested for transamination and β-elimination activities *in vitro*. Western blot analysis confirmed that most mutants exhibited expression levels comparable with the wildtype protein. Notably, H279F, R398A, N185Q, F125H, and W18H mutants showed approximately two-fold higher expression (Supplementary Figure S1). Among the W18 variants, W18H displayed the highest expression, while W18L showed the lowest.

### Mutation-driven modulation of hKYAT1 activity: divergent effects on aromatic amino acids and kynurenine

**Figure 2:**
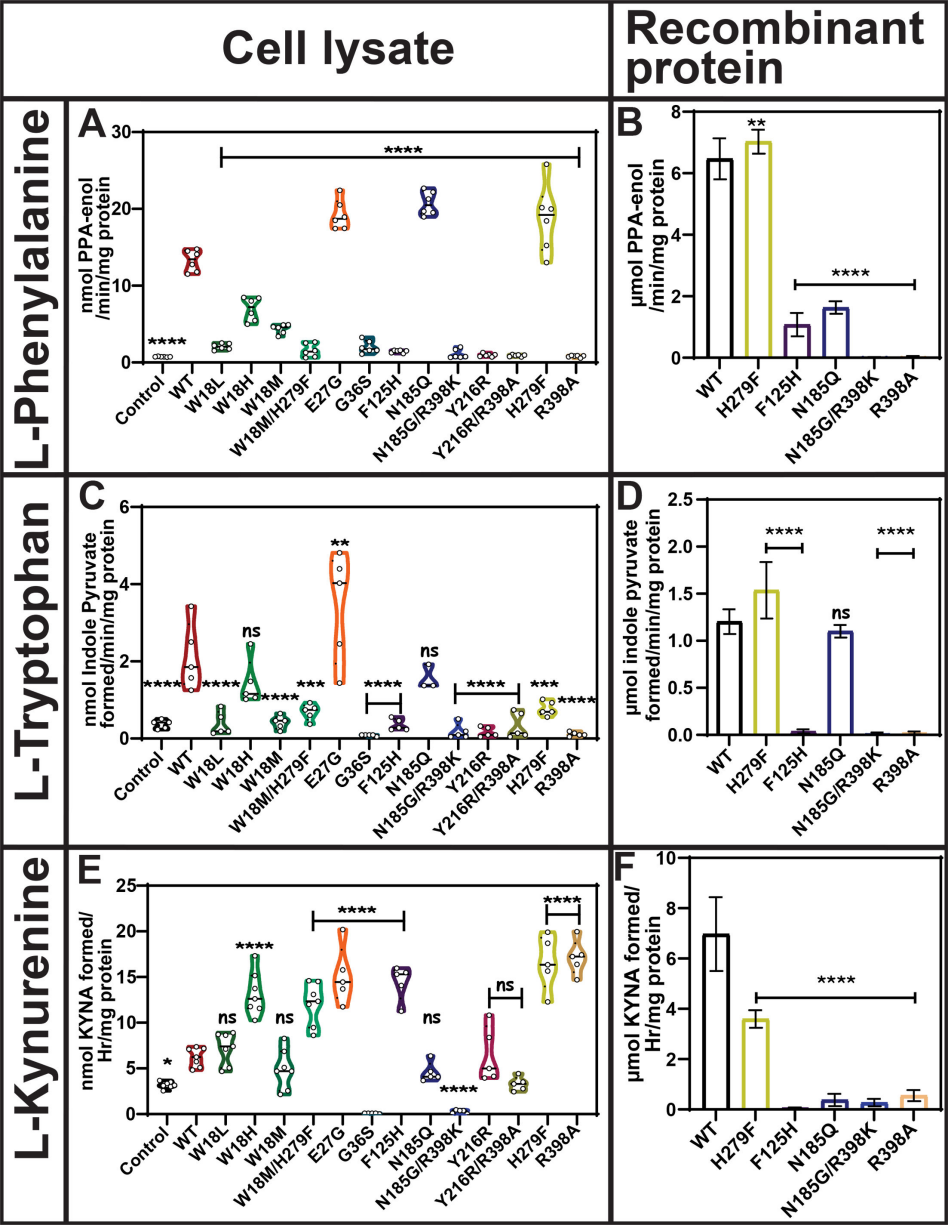
H279 mutation in the substrate stabilization site alters hKYAT1 transamination activity for aromatic amino acids and kynurenine. Transamination activity of thirteen hKYAT1 mutants was evaluated for l-Phe (20 mM), l-Trp (3 mM), and Kyn (3 mM) using whole-cell lysates with 20 µg of crude protein (*n* = 5–7) (**A**, **C**, and **E**). Bars are presented in the order: control (empty vector), wildtype hKYAT1, and followed by individual mutants. Five recombinant hKYAT1 mutants (200 ng protein) were analyzed separately (*n* = 5–8) (**B**, **D**, and **F**). Bars are presented in the order: wildtype hKYAT1, and followed by individual mutants. Statistical significance was determined using one-way ANOVA with a 95% confidence interval, followed by Dunnett’s multiple comparisons test (mean ± sd ns = not significant, *P<0.05, **P<0.01, ***P<0.001, and ****P<0.0001 compared with wildtype hKYAT1 overexpressed lysate/wildtype hKYAT1 recombinant protein). Each amino acid assay was performed in five to eight independent experiments. hKYAT1, human kynurenine aminotransferase 1.

Residue substitutions in the catalytic site (helix a1, E27G), ligand-binding site (N185Q), and substrate stabilization site (H279F) resulted in a 1.5- to 2-fold increase in the transamination of l-Phe as compared with wildtype hKYAT1 lysate (Figure 2A). In contrast, other mutants significantly reduced l-Phe transamination (Figure 2A). The H279F substitution significantly increased the transamination activity of the recombinant hKYAT1, corroborating the results from cell lysate assays (Figure 2B). Interestingly, recombinant N185Q-hKYAT1 displayed significantly reduced transamination activity (Figure 2B), differing from the increased activity observed in cell lysates (Figure 2A). Substitutions at residue 398 (R398A- and N185G/R398K) abolished the transamination capacity of hKYAT1 for l-Phe (Figure 2B), as further supported by enzyme kinetics showing H279F with a higher *K*_cat_/*K*_M_ ratio compared with wildtype (Supplementary Figure 1A). Aside from the observed dichotomy for the N185Q mutant, results from both cell lysate and recombinant protein assays were consistent. Additionally, the *K*_M_ and *K*_cat_ values for the five mutated enzyme variants support these findings (Supplementary Figure 1A, Table 1). In conclusion, while some mutations significantly enhance transaminase activity, others completely abolish it, underscoring the critical roles of the selected residues in l-Phe transamination.

**Table 1:** Michaelis–Menten kinetic parameters for transamination and β-elimination of the different recombinant mutant proteins with various amino acid substrates

Amino acid	Mutant KYAT1	*K*_M_ (mM)	*K*_cat_ (s^-1^)	*K*_cat_/*K*_M_ (s^-1^ mM^-1^)
l-Phe	WT	1.7 ± 0.3	10099 ± 210	5854 ± 1116
	H279F	1.5 ± 0.4	9428 ± 226	6118 ± 1453
	F125H	9.3 ± 1.4	1647 ± 61	178 ± 27
	N185Q	1.1 ± 0.4	4069 ± 112	3669 ± 1266
	N185G/R398K	7.8 ± 10.2	102 ± 31	13 ± 18
	R398A	ND	ND	ND
l-Trp	WT	0.9 ± 0.2	3881 ± 270	4511 ± 1301
	H279F	1.2 ± 0.1	6540 ± 189	5662 ± 576
	F125H	7.3 ± 2.8	1660 ± 419	229 ± 105
	N185Q	1.2 ± 0.1	3500 ± 85	2951 ± 250
	N185G/R398K	1.5 ± 1.8	340 ± 139	228 ± 290
	R398A	1.2 ± 0.4	297 ± 30	248 ± 86
l-Kyn	WT	1.8 ± 0.5	21469 ± 2057	12254 ± 3391
	H279F	5.3 ± 4.6	14717 ± 7656	2794 ± 2835
	F125H	ND	ND	ND
	N185Q	0.8 ± 1.6	910 ± 417	1096 ± 2121
	N185G/R398K	ND	ND	ND
	R398A	ND	ND	ND
MSC	WT	15.7 ± 5.4	12840 ± 2973	818 ± 339
	H279F	0.8 ± 0.2	5096 ± 191	6377 ± 1192
	F125H	61.2 ± 188	12056 ± 32726	197 ± 809
	N185Q	3.1 ± 2.0	1081 ± 262	353 ± 247
	N185G/R398K	ND	ND	ND
	R398A	1.4 ± 0.9	841 ± 147	600 ± 407
SeMet	WT	17.9 ± 6.8	2668 ± 645	149 ± 67
	H279F	119 ± 7	12739 ± 20546	107 ± 256
	F125H	4.4 ± 2.6	826 ± 190	188 ± 118
	N185Q	ND	ND	ND
	N185G/R398K	0.7 ± 0.6	456 ± 82	687 ± 688
	R398A	0.01 ± 0.2	516 ± 53	1.1*10^5^ ± 5.0*10^6^
β-elimination activity				
MSC	WT	6.2 ± 2.1	11344 ± 1852	1829 ± 299
	H279F	7.9 ± 1.8	13695 ± 1618	1726 ± 204
	F125H	10.9 ± 7.5	6240 ± 2574	572 ± 236
	N185Q	52.9 ± 41.5	50599 ± 34313	957 ± 649
	N185G/R398K	ND	ND	ND
	R398A	20.0 ± 15.1	19766 ± 10729	986 ± 535
SeMet	WT	0.01 ± 0.2	7744 ± 737	1.1*10^6^ ± 1.1*10^5^
	H279F	0.7 ± 0.2	2963 ± 184	4320 ± 268
	F125H	0.7 ± 0.6	4781 ± 731	6915 ± 1057
	N185Q	0.3 ± 0.1	4414 ± 214	18141 ± 878
	N185G/R398K	ND	ND	ND
	R398A	ND	ND	ND

Tryptophan is another important aromatic amino acid substrate for hKYAT1. Most of the tested mutants either abolished or reduced hKYAT1’s transamination activity toward l-Trp, except for the E27G mutant, which resulted in a two-fold increase in activity compared with wildtype hKYAT1 lysate (Figure 2C). Notably, the W18H and N185Q mutants did not affect l-Trp transamination in cell lysates as compared with the wildtype (Figure 2C). As observed for l-Phe, the results from recombinant proteins both confirmed and contrasted with the cell lysate data (Figure 2C and D). While the H279F variant displayed a 1.5-fold increase in activity compared with wildtype hKYAT1 in recombinant protein assays, no significant difference was observed in the cell lysates (Figure 2C and D). Interestingly, the recombinant protein results showed similar mutational effects for both l-Phe and l-Trp (Figure 2B), aligning well with the *K*_M_ and *K*_cat_ values for these substrates (Supplementary Figure S1B, Table 1). The same set of mutants was tested with dl-Tyr (Supplementary Figure 2A & 2B). The E27G mutant exhibited comparable transamination activity with wildtype hKYAT1 for dl-Tyr, while all other mutants reduced hKYAT1’s activity toward l-Tyr. These findings were confirmed by recombinant assays, where most mutants abolished dl-Tyr transamination, except for H279F (Supplementary Figure 2A & 2B).

A major function of hKYAT1 is the transamination of l-Kyn to Kyna. Interestingly, mutants W18H, W18M/H279F, E27G, F125H, H279F, and R398A increased the enzymatic activity of hKYAT1 toward l-Kyn by two- to four-fold in cell lysates (Figure 2E), a pattern that contrasts with the activity observed for l-Phe and l-Trp (Figure 2A, C and D). However, all five tested recombinant mutants either reduced or abolished l-Kyna production as compared with the wildtype (Figure 2F). The *K*_M_ and *K*_cat_ values for the mutant recombinant hKYAT1 variants toward l-Kyn also differed significantly from those for l-Phe and l-Trp. Specifically, all five recombinant mutants, including H279F, exhibited lower *K*_cat_/*K*_M_ ratios as compared with the wildtype, indicating lower catalytic efficiency for l-Kyn transamination (Supplementary Figures S1A, S1B & S1C, Table 1).

Overall, our results indicate that hKYAT1 employs different key residues for transamination depending on the substrate, whether it be aromatic amino acids or kynurenine. Interestingly, the expression levels of the recombinant proteins did not directly correlate with hKYAT1 enzymatic activity (Supplementary Figure 3A & 3B). For instance, while H279F-hKYAT1 exhibited significantly higher transamination activity toward l-Phe, the F125H- and R398A-hKYAT1 variants showed no transamination capacity for these substrates. Even though the expression levels of F125H- and R398A-hKYAT1 were twice as high as wildtype-hKYAT1, they displayed no transamination activity across a broad range of tested substrates.

### Mutational analysis and substrate preferences of hKYAT1 highlight key residues in transamination activity

**Figure 3:**
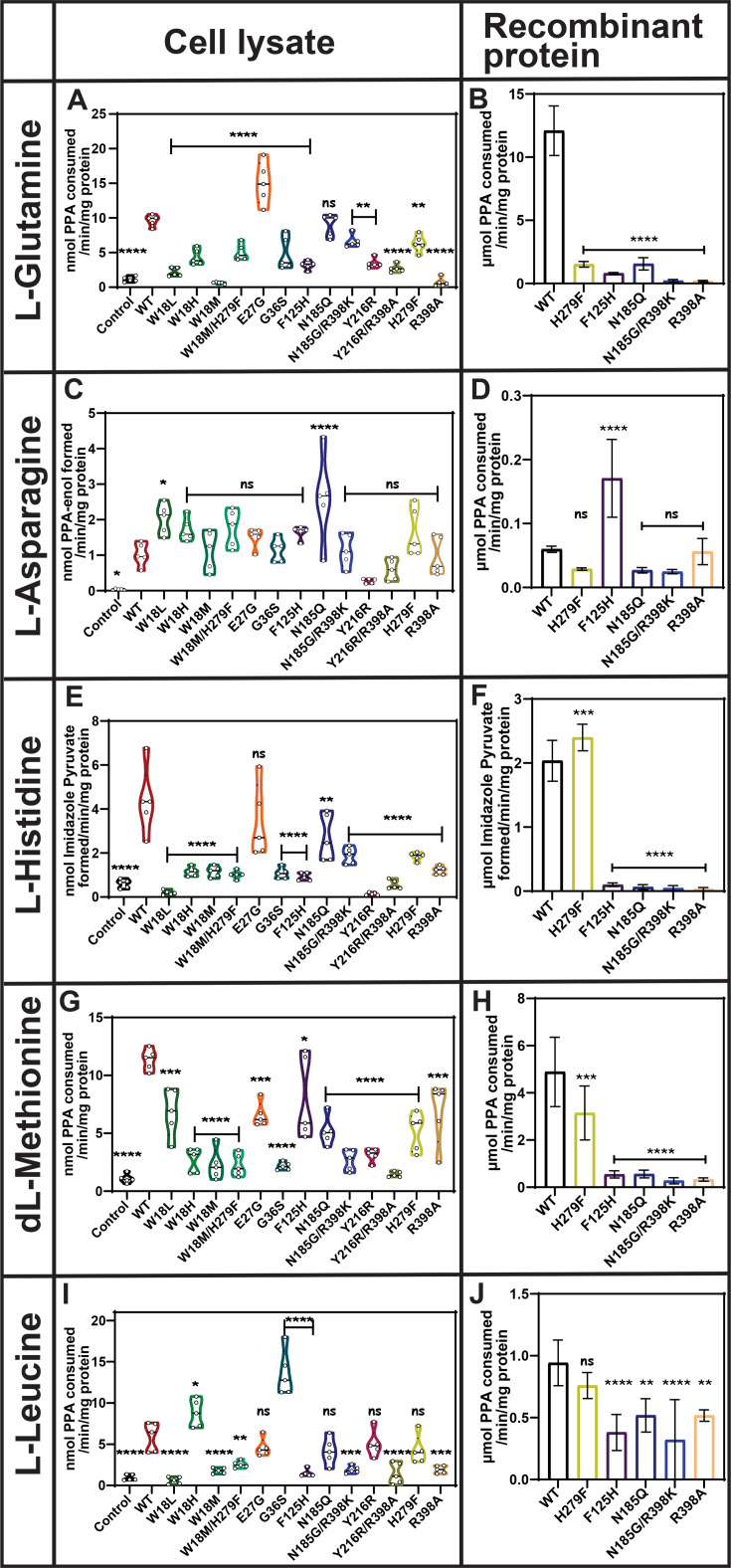
Impact of mutations on transamination of non-aromatic amino acids. Transamination activity of 13 hKYAT1 mutant variants was tested for amidic l-Gln (3 mM), basic l-His (5 mM), acidic l-Asp (3 mM), sulfur-containing dl-Met (3 mM), and aliphatic l-Leu (3 mM) substrates. Panels **A, C, E, G,** and **I** show results from cell lysates containing 20 µg of crude protein (*n* = 5–7), while panels **B, D, F, H,** and **J** present data from wildtype and five recombinant mutant proteins with 200 ng of recombinant protein (*n* = 5–8). (**A, C, E, G,** and **I** ) Bars are presented in the order: control (empty vector), wildtype hKYAT1, and followed by individual mutants. (**B, D, F, H,** and **J**) Bars are presented in the order: wildtype hKYAT1, and followed by individual mutants. Statistical significance was determined using one-way ANOVA with a 95% confidence interval, followed by Dunnett’s multiple comparisons test (mean ± sd ns = not significant, *P<0.05, **P<0.01, ***P<0.001, and ****P<0.0001 compared with wildtype hKYAT1 overexpressed lysate/ wildtype hKYAT1 recombinant protein). Each amino acid assay was performed in five to eight independent experiments. hKYAT1, human kynurenine aminotransferase 1.

Amidic amino acids are essential for cancer cell survival as they can be converted into their respective α-keto acids via transamination. Notably, essential α-keto acids, such as α-ketoglutarate and oxaloacetate, which are derived from the transamination of glutamine and asparagine, respectively, play crucial roles in cell survival as key components of the TCA cycle. Overexpression of hKYAT1 in HEPG2 cells resulted in a five-fold increase in l-Gln transamination compared with control cell lysates (Figure 3). However, mutations of residues in the ligand recognition site (W18), stabilization site (F125, Y216, and R398), and substrate stabilization site (H279) significantly reduced or completely abrogated enzymatic activity toward l-Gln. In contrast, mutations at the catalytic site (E27) increased hKYAT1 transamination activity toward l-Gln, while mutation of N185 in the ligand-binding site showed activity as comparable with wildtype hKYAT1 (Figure 3A). These findings were consistent with the results obtained using recombinant proteins (Figure 3B). Furthermore, transamination activity toward l-asparagine (l-Asn) was minimal across all tested mutants using both cell lysates and recombinant proteins. However, mutation at W18L and N185Q exhibited a notable increase in transamination activity as compared with wildtype hKYAT1 cell lysates (Figure 3C), and interestingly, recombinant F125H-hKYAT1 displayed a significant increase in transamination activity compared with wildtype hKYAT1 (Figure 3D). Similarly, hKYAT1 exhibited minimal activity with the acidic amino acid l-aspartic acid (l-Asp), a finding consistent across both cell lysate and recombinant protein assays (Supplementary Figure S3C, & 3D). While some mutations suggested a modest increase in transamination activity in cell lysates, recombinant protein assays confirmed that l-Asn and l-Asp are not major substrates to hKYAT1 (Figure 3D, Supplementary Figure S3D). Most hKYAT1 mutants displayed reduced transamination activity toward l-histidine (l-His) in cell lysate assays, except for the E27G mutant, which showed enhanced activity (Figure 3E). Conversely, recombinant H279F displayed a significant increase in l-His transamination compared with wildtype hKYAT1, while all other mutants abrogated transamination activity (Figure 3F). These results underscore the importance of residue H279 in l-His transamination (Figure 3E and F).

Wildtype hKYAT1 overexpression had a profound impact on the transamination of the sulfur-containing amino acid methionine, resulting in a nine-fold increase in transamination activity as compared with control lysates (Figure 3G). Interestingly, all tested mutants significantly reduced hKYAT1’s transamination activity toward dl-Met in cell lysates (Figure 3G). This was further confirmed in recombinant protein assays, where all mutants, except H279F, partially abrogated dl-Met transamination (Figure 3H). Additionally, hKYAT1 displayed minimal activity with l-cystine (l-Cyss), a result corroborated by both cell lysate and recombinant protein assays (Supplementary Figure S3E, &3F). hKYAT1 efficiently transaminated the aliphatic amino acid l-Leu, exhibiting a seven-fold increase in transamination activity as compared with control lysates (Figure 3I). Notably, the W18H and G36S mutants significantly increased transamination activity toward l-Leu compared with wildtype hKYAT1 (Figure 3I). In contrast, mutation of residue W18 to leucine (W18L) or methionine (W18M) reduced transamination activity toward l-Leu (Figure 3I). Several other mutants, including R398A, N185G/R398K, and Y216R/R398A, also reduced or abrogated l-Leu transamination, highlighting the importance of R398 in l-Leu transamination (Figure 3I). These results were validated by recombinant protein assays (Figure 3J). Additionally, we tested smaller hydrophobic substrates such as l-alanine (l-Ala), proline (Pro), and Gly. While some transamination activity was detected, these residues are not primary substrates for hKYAT1 (Supplementary Figure S**3G,3H, 3I, 3J, 3K** **, &** **3L**). In conclusion, our results demonstrate that Gln, Met, His, and Leu are the most favorable substrates for hKYAT1, while Asn, Asp, Cyss, Ala, Pro, and glycine (Gly) are the least preferred substrates.

### Functional characterization of hKYAT1 mutants: enhanced activity with MSC compared with SeMet

**Figure 4:**
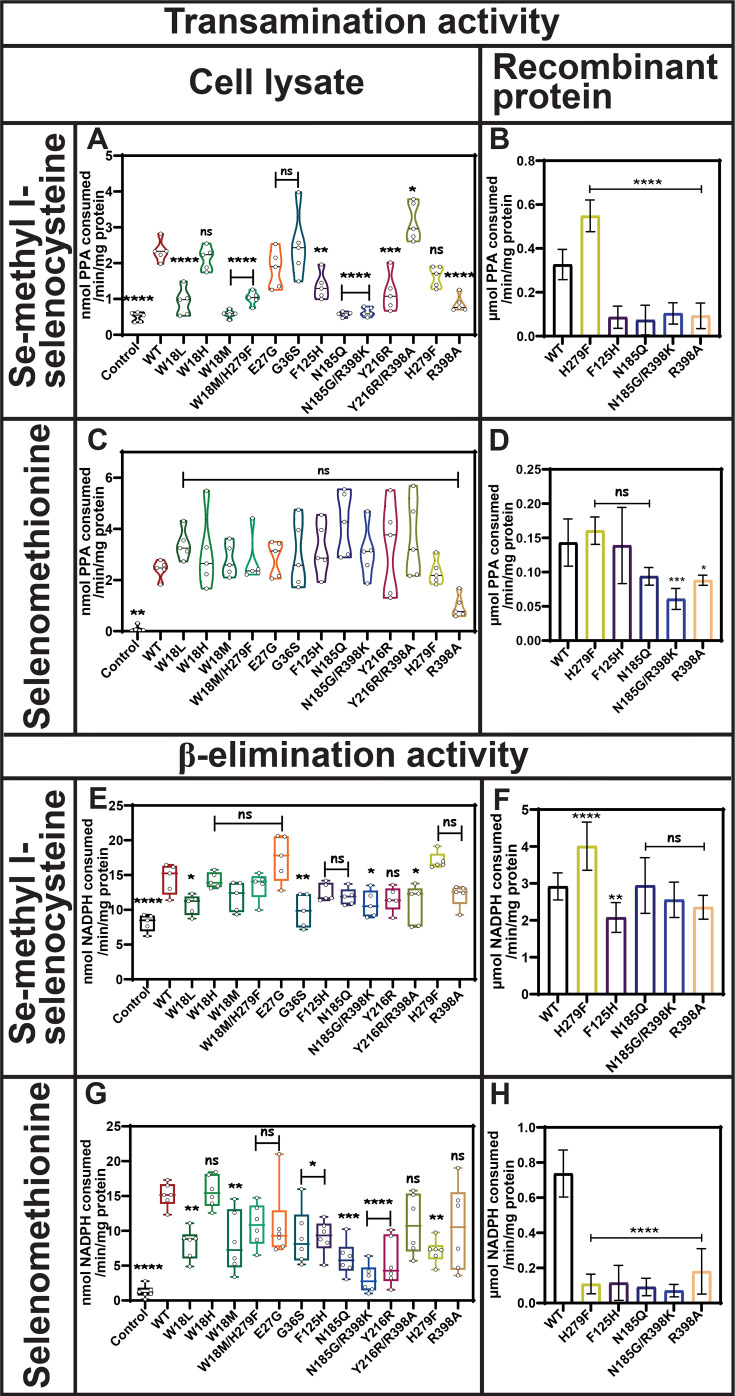
Mutation at H279 modulates MSC metabolism without affecting SeMet activity. Transamination efficacy of 13 mutated hKYAT1 variants was assessed for Se-conjugated compounds, MSC (5 mM) and SeMet (5 mM). Panels **A**, **C** show activity with cell lysates containing 20 µg of crude protein, while panels (*n* = 5–7) and **B, D** present data for five recombinant mutant proteins (*n* = 5–8). β-elimination activity was tested in panels **E**, **G** with cell lysates (20 µg of crude protein) (*n* = 5–7), and in panels **F**, **H** with recombinant mutant proteins (200 ng) (*n* = 5–8). (**A, C, E, and G**) Bars are presented in the order: control (empty vector), wildtype hKYAT1, and followed by individual mutants. (**B, D, F, and H**) Bars are presented in the order: wildtype hKYAT1, and followed by individual mutants. Statistical significance was determined using one-way ANOVA with a 95% confidence interval, followed by Dunnett’s multiple comparisons test (mean ± sd ns = not significant, *P<0.05, **P<0.01, ***P<0.001, and ****P<0.0001 compared with wildtype hKYAT1 overexpressed lysate/ wildtype hKYAT1 recombinant protein). Each amino acid assay was performed in five to eight independent experiments. hKYAT1, human kynurenine aminotransferase 1; MSC, methylselenocysteine.

KYAT1 transaminates Se-conjugated compounds, including MSC and SeMet, converting them to β-methylselenopyruvate (MSP) and α-keto-γ-methylselenobutyrate (KMSB), respectively. The Y216R/R398A mutant displayed elevated MSC transamination activity compared with wildtype, while mutants such as W18H, E27G, G36S, and H279F showed comparable MSC transamination activity with wildtype hKYAT1 (Figure 4A). Substitution of the aromatic residue at position 279 (H279F) resulted in a two-fold increase in MSC transamination activity in recombinant proteins as compared with wildtype (Figure 4B). This increased transamination activity in the H279F mutant was reflected in the MSC kinetics, with *K*_M_ and *K*_cat_ values of 15.7 ± 5.4 and 0.8 ± 0.15 mM, and 12840 ± 2973 and 5096 ± 191 s^-1^ for wildtype and H279F, respectively (Supplementary Figure S1D, Table 1). In contrast, none of the tested hKYAT1 mutants showed any significant transamination activity with SeMet, compared with wildtype hKYAT1 cell lysate, although hKYAT1-overexpressed cell lysates (wildtype or mutant) demonstrated significant transamination activity as compared with control cell lysates (Figure 4C). These results suggest that hKYAT1 preferentially transaminates MSC over SeMet (Figure 4D). The SeMet kinetics for wildtype and mutant hKYAT1 further support this preference, showing *K*_M_ and *K*_cat_ values of 17.9 ± 6.8 and 119.6 ± 7.0 mM, and 2668 ± 645 and 12739 ± 20546 s^-1^ for wildtype hKYAT1 and H279F, respectively (Supplementary Figure S1D , Table 1).

In addition to cystine S-conjugates, hKYAT1 metabolizes Se-conjugates including MSC and SeMet, exhibiting β-elimination activity that cleaves MSC and SeMet into MS. This is attributed to the weaker C-Se bond compared with the C-S bond. Our data demonstrated that hKYAT1 induction enhanced β-elimination activity two-fold with MSC. Mutants such as W18L, G36S, N185G/R398K, and Y216R/R398A decreased β-elimination activity as compared with wildtype in the cell lysates (Figure 4E), while all other mutants showed similar β-elimination activity to wildtype in cell lysates (Figure 4E). In recombinant proteins, the H279F mutant exhibited a significant increase in β-elimination activity compared with wildtype (Figure 4F). This elevated β-elimination activity for both wildtype and the H279F mutant in recombinant proteins was reflected in their *K*_M_ and *K*_cat_ values (Supplementary Figure S1F, Table 1).

SeMet was also tested for β-elimination activity in both wildtype and mutant hKYAT1. We observed elevated β-elimination activity for SeMet in hKYAT1-overexpressing cell lysates compared with control lysates. Different hKYAT1 mutants exhibited varying abilities to cleave SeMet into MS via β-elimination activity (Figure 4G). Results obtained with recombinant mutant and wildtype enzymes indicated that SeMet is not a preferred β-elimination substrate for hKYAT1 compared with MSC (Figure 4H)**.** This preference was further confirmed by the *K*_M_, *K*_cat_, and *K*_cat_/*K*_M_ values for SeMet (Supplementary Figure S1G, Table 1). A comparison of β-elimination activity between HEPG2 cell lysates and recombinant proteins for MSC and SeMet confirmed that MSC is favored as a substrate of hKYAT1 over SeMet.

## Discussion

Human KYAT1 plays a crucial role in recovering α-keto acid analogs from several essential amino acids through transamination reactions [8,25]. It is a key enzyme in the metabolism of the essential amino acid tryptophan, serving as the primary source for *de novo* nicotinamide adenine dinucleotide (NAD) biosynthesis [16]. It transfers an amino group from kynurenine (an amino group donor in the Trp pathway) to α-ketoglutarate (an amino group acceptor), producing anthranilic acid and glutamate. The activity of hKYAT1, which exhibits broad substrate specificity, is regulated by multiple factors, including substrate availability, cofactors, and cellular energy status. As such, this crucial enzyme is involved in a range of physiological and pathological processes, including neurodegeneration, inflammation, and cancer [26,27].

The presence of aromatic residues in the ligand-binding region of hKYAT1 is crucial for substrate recognition [22,24]. Mutation analysis of catalytic, ligand-binding, and substrate-stabilization sites is therefore essential to understand the enzyme’s specificity toward its broad range of substrates. In this study, we identified and mutated several key residues in hKYAT1 and systematically characterized the effects of each substitution on a large array of amino acid substrates. It is well established that hKYAT1 prefers different α-keto acids (amino group acceptors) depending on the substrate [8]. For example, hKYAT1 utilizes α-keto-γ-methylthiobutyric acid (KMB) and 2-keto-butyric acid (KBA) for the transamination of phenylalanine and kynurenine, respectively, while α-ketoglutarate (αKG) and KMB are required for β-elimination activity with MSC. We carefully evaluated various α-keto acids (KMB, KBA, αKG, pyruvate, phenylpyruvate (PPA), and glyoxylate) for their transamination activity with different substrates. Our results demonstrated that KMB, KBA, and PPA were the primary α-keto acid (amino group acceptors) for hKYAT1, with αKG showing the least activity, which is supported by the previous studies [8,28].

In addition, hKYAT1 is highly efficient at metabolizing α-keto acids into their corresponding amino acids, catalyzing reactions including the conversion of phenylpyruvate (PPA) to l-Phe, and indole-3-pyruvate (IPA) to l-Trp [3,29]. Previous studies have shown that hKYAT1 prefers transamination of IPA to l-Trp over transamination of l-Trp to IPA [3]. These findings align with our results, which demonstrated increased transamination of aromatic amino acids like Phe and Trp. Aromatic amino acids are essential for various physiological functions, including quenching reactive oxygen species, providing neuroprotection, and facilitating signal transmission [10,30,31]. Defects in their metabolism can have significant repercussions on human health [32].

In this study, we demonstrated that mutations in the substrate binding site (H279) and catalytic site (E27) significantly modulated the transamination activity of hKYAT1 for all aromatic amino acids in experiments performed in cell lysates. To our knowledge, the only reported naturally occurring mutant variant is the E61G mutation in rat KYAT1 (rKYAT1). It should be noted that residue E61 in rKYAT1 corresponds to E27 in hKYAT1 [33]. Rossi et al. previously proposed that E27 plays a critical role in the catalytic site of hKYAT1, where its mutation reduced enzyme activity and caused hypertension in rats [9,33]. However, we observed the opposite, since E27 increased the activity in this study. This could be due to greater flexibility in the α1-helix, potentially enhancing the adaptability of the active site for larger substrates [34]. The E27G mutant increased enzymatic activity across most substrates, particularly Gln, but showed only moderate activity with Asp. These trends suggest a possible role for Gln-derived metabolites, like αKG, in fueling the TCA cycle and maintaining anaplerosis; however, this interpretation is based on lysate data and would require recombinant validation for confirmation [31,35]. Comparison of the crystal structure of hKYAT1 in complex with different substrates revealed that the lateral movement of the α1-helix is necessary for accommodating larger ligands, but not small ones [22]. Residue H279 forms a hydrogen bond with bound substrates and plays a key role in stabilizing ligands, as demonstrated in the crystal structure of hKYAT1 [24]. Our findings show that the recombinant H279F mutant significantly increased hKYAT1 activity toward Phe, Trp, His, and MSC but reduced activity toward Tyr, Gln, Met, Gly, Cyss, and Kyn. Although the E27G mutant consistently exhibited increased activity across several substrates in lysate-based assays, it was not included in the recombinant protein analysis due to prioritization of functionally distinct variants. Given its position in the α1-helix and potential role in enhancing structural flexibility, E27G remains an intriguing candidate for future structural and enzymatic characterization.

We observed in a few cases contradictory transamination activities between cell lysates and recombinant proteins. This variation could be attributed to the presence of other enzymes present in the whole-cell lysates, such as ω-amidase, tyrosine transaminase (TAT), and cytosolic malate dehydrogenase (MDH1), which are involved in the Trp salvage pathway [3] or due to changes in expression levels of the mutated protein. Indeed, it is known that mutated proteins can have an expression level different from that of the wildtype protein [36]. For example, in HCM patients heterozygous for mutations in the MYH7 gene encoding for the β-myosin heavy chain, expression of the mutant and the wildtype allele can be unequal and fractions of mutant and wildtype mRNA and protein significantly deviate from 1:1 [37]. Even synonymous mutation of the gene can change the expression of the protein due to the position and context of codons in the genes, recombination rates, mRNA folding and stability, tRNA abundance, and so on [38]. There is growing evidence that some synonymous mutations can cause disease through the disruption of different processes of protein production [39]. Additionally, since hKYAT1 functions as a homodimer, mutant KYAT1 subunits may heterodimerize with endogenous wildtype hKYAT1, potentially affecting transamination activity in lysate assays. To ensure accurate interpretation of mutation-specific effects, we complemented lysate-based assays with purified recombinant proteins. This approach provided a controlled system to assess catalytic activity without interference from endogenous KYAT1.

Residue F125 is conserved across mammalian KYAT proteins, but in human KYAT2 and KYAT3, F125 is replaced by Tyr. In KYAT2, the Y125F mutant resulted in a 20-fold reduction in β-lytic activity [40]. Our findings indicate that the F125H mutant significantly affected substrate binding across all tested amino acid substrates. Residue W18, located at the hinge of the α1-helix, plays a critical role in ligand recognition and stabilization [22]. Mutation at this residue inactivated hKYAT1 with most amino acids except Asp; notably, hKYAT1 has minimal activity toward Asp. Interestingly, the W18H mutant exhibited increased transamination activity for Kyn and Leu, although expression levels were double that of the wildtype (supplementary Figure 3). Residue G36, located at the entrance of the substrate channel, plays a critical role, as demonstrated by the G36S mutant, which showed two-fold higher transamination activity for Leu compared with the wildtype. Mutation at N185 and R398, both key residues in the ligand-binding regions [22,24], revealed additional insights. The N185Q/G mutants exhibited moderate activity for most substrates, with notably higher activity for l-Phe. In contrast, the R398A and Y216R mutant resulted in complete inactivation of hKYAT1, confirming the critical role of these residues in catalysis and any residual changes at this site completely destabilize the enzyme.

Transamination of Kyn to Kyna is a key step in the KP, which plays an important role in inflammatory responses, immunoregulation, and psychiatric disorders [41]. Kyna is considered neuroprotective, suppressing several inflammatory pathways and playing a significant role in immune responses by activating the aryl hydrocarbon receptor (AhR) [41]. Interestingly, while the transamination of Kyn increased in hKYAT1 mutants in cell lysates, recombinant hKYAT1 mutants showed the opposite trend. This discrepancy suggests that Kyna production may not occur under recombinant conditions and could require additional factors, such as cofactors or other enzymes, to drive the reaction or due to higher expression levels of the mutated protein, notably W18H, F125H, N185Q, H279F, and R398A. It has been reported that Kyna does not cross the blood–brain barrier, whereas Kyn does, suggesting that Kyna is synthesized in the brain [42].

Our results demonstrated significantly higher transamination and β-elimination activity for the H279F mutants with MSC, but not with SeMet. The transamination and β-elimination of MSC result in the formation of β-MSP and methylselenol (MS), respectively [42]. In contrast, the transamination and β-elimination of SeMet generate KMSB and MS, respectively [43]. Both MSC and SeMet metabolites are known to modulate tumor cell growth and possess chemopreventive activities [43,44]. Specifically, MSP has been reported to inhibit histone deacetylases (HDACs), as it structurally resembles butyrate, a potent HDAC inhibitor [43]. Additionally, the β-elimination products of MSC and SeMet can alter the redox balance, leading to changes in cell signaling pathways and the induction of proapoptotic genes in various cancers [45]. Zeng et al. (2006) reported that MS down-regulates the BCL2-related protein A1 (BCL2A1), leading to increased cytochrome C release into the cytoplasm and initiation of apoptotic events [46,47]. Moreover, MSC has been shown to induce apoptosis by activating multiple kinase signaling pathways and by participating in the regulation of the NF-kB signaling pathway during inflammatory responses [48]. In our study, elevated β-elimination activity with MSC was observed in both the E27G and H279F mutants in cell lysates. Furthermore, the recombinant H279F mutant exhibited higher β-elimination activity with MSC compared with the wildtype. In contrast, the other tested recombinant proteins displayed β-elimination activity similar to the wildtype, with the exception of F125H. Interestingly, only the wildtype enzyme showed moderate β-elimination activity with SeMet, further confirming that KYAT1 preferentially utilizes MSC as a substrate over SeMet, consistent with previous findings [49].

Despite transfecting equal quantities of mutant hKYAT1 plasmids into HEPG2 cells, we observed variability in expression levels among the mutants (Supplementary Figure S3A & 3B). Interestingly, the effect of these mutations appeared to be substrate-dependent. For example, while the H279F, R398A, and F125H mutants showed a two-fold increase in protein expression as compared with wildtype, this did not directly correlate with enzyme activity. Specifically, the H279F mutant displayed higher transamination activity toward l-Phe, whereas the R398A and F125H mutants showed minimal transamination activity toward l-Phe. Additionally, despite its expression being twice as high as wildtype, the R398A mutant showed no significant activity toward several amino acid substrates (Supplementary Figure S3A & 3B). These observations were further validated in recombinant hKYAT1 assays, where an equal quantity of hKYAT1 mutants (H279F, R398A, and F125H) was used in all transamination and β-elimination assays. This underscores the importance of substrate specificity and the differential impact of mutations on hKYAT1 enzyme activity.

In conclusion, our study demonstrates that the transamination and β-elimination activity of hKYAT1 mutants vary depending on the substrate and the residues involved. We identified two key mutants, H279F and E27G, that significantly enhanced transamination activity toward several hKYAT1-preferred substrates, including MSC. These findings highlight the possibility of tuning hKYAT1 function through targeted mutagenesis to optimize the metabolism of selenoamino acids with antineoplastic potential. While these results are promising, further investigation is necessary to understand the structural and physiological implications of these mutations *in vivo*. Continued exploration of KYAT1 variants in disease-relevant models will be critical to evaluate their potential utility in therapeutic strategies involving selenium metabolism.

## Methods

### Chemicals and reagents

l-Trp, l-Kyn, l-Leu, l-Ala, l-Gln, l-Cyss, l-His, l-Asn, l-Asp, phenylpyruvic acid (PPA), KMB, KBA, Se-MSC, dimethyl-2-oxoglutarate (α-KG), Gly, Pro, l-Phe, dl-Met, dl-Tyr, 2-amino-2-methyl-1,3-propanediol, PLP, phenylmethanesulfonyl fluoride (PMSF), RIPA buffer, protease inhibitor cocktail mix, N-N-dimethyl formamide, potassium dihydrogen phosphate, di-potassium hydrogen phosphate, EDTA, sodium arsenate dibasic heptahydrate, and sodium hydroxide were purchased from Sigma-Aldrich (St. Louis, MO, U.S.A.). l-Selenomethionine was purchased from Santa Cruz (Dallas, TX, U.S.A.). NADPH was purchased from Acros Organics (Geel, Belgium). Lipofectamine 3000 was purchased from Invitrogen (Camarillo, CA, U.S.A.). Page Ruler Plus Prestained protein ladder was purchased from Thermo Fisher Scientific (Rockford, IL, U.S.A.). Plasmid pEGFP-N1 (Clontech, Takara Bio Inc., Mountain View, CA, U.S.A.) was kindly provided by Dr. Gildert Lauter and Dr. Peter Swoboda, Department of Biosciences and Nutrition, Karolinska Institute. Mammalian TrxR1 was purchased from Sigma-Aldrich (Darmstadt, Germany).

### Cell culture and growth conditions

HEPG2 cells were purchased from ATCC (Wesel, Germany) and maintained in EMEM (ATCC) supplemented with 10% heat-inactivated fetal bovine serum (FBS; Gibco, Paisley, UK) under 5% CO_2_ at 37°C without antibiotics. Cells were regularly tested for mycoplasma contamination using MycoAlert (Lonza, Boston, MA, U.S.A.). Cell counting was performed using a TC 20^TM^ automated cell counter (Bio-Rad, Portland, ME, U.S.A.).

### Cloning and cellular overexpression of hKYAT1 and mutant variants

The full-length human KYAT1 (hKYAT1) coding sequence was amplified from cDNA and cloned into the mammalian expression vector pEGFP-N1 (Clontech, Takara Bio Inc., CA, U.S.A.). The human PGK promoter and puromycin resistance gene sequences were amplified from pLKO.1 (Sigma Aldrich, Darmstadt, Germany) and inserted into the pEGFP-N1 backbone containing the hKYAT1 expression vector. Point mutations were introduced in this hKYAT1 backbone, and all mutants were confirmed by Sanger sequencing. An empty vector was created by inserting the PCR-amplified puromycin resistance gene expression cassette into pEGFP-N1. Protein sequences used in this study are listed in Supplementary Table S1.

### Cloning, expression, and purification of recombinant hKYAT1 and mutated variants

The production and isolation of recombinant proteins were carried out using full-length hKYAT1 cDNA, which was amplified and cloned into the pET-23a expression vector (Novagen, Cambridge, UK) using the ligation-independent ‘Fast Cloning’ method [50]. All mutants were confirmed by Sanger sequencing. The plasmids were transformed into T7 Express Competent E. coli (New England Biolabs, U.S.A.), and overnight cultures were grown in Luria–Bertani broth (LB) medium containing 100 mg/ml ampicillin at 37°C, shaking at 120 rpm, and until OD600 reached 0.8. After cooling the cultures for 30 minutes at 4°C, protein expression was induced with 0.5 mM IPTG and grown overnight at 16°C. Harvested cells were stored at −20°C until use. All protein purification steps were performed at 4°C. Cell pellets were lysed in PBS buffer and subjected to ultrasonication. After centrifugation at 60,000×g for 30 min, protein in the supernatant was affinity purified using a HisTrap™ HP column (Cytiva, Uppsala, Sweden). Proteins were concentrated, and size exclusion chromatography was performed using a Superdex 200 increase 10/300 column (Cytiva, Uppsala, Sweden). Purified proteins were flash frozen in liquid nitrogen and stored at −80°C.

### Overexpression of wildtype and mutated hKYAT1 variants in HEPG2 cells

HEPG2 cells were seeded at 400 cells/mm^2^ in six-well plates. Twenty hours later, cells were transfected with 2 µg of plasmids DNA encoding wildtype or mutant hKYAT1 using Lipofectamine 3000 (Invitrogen, U.S.A.). An empty vector served as a negative control. Cells were harvested 48 h post-transfection, and protein expression was verified by Western blot. Whole-cell lysates were used for subsequent enzyme assays.

### Determination of protein concentration

Samples were lysed on ice for 30 min in RIPA buffer containing 1 mM PMSF and 1% protease inhibitor cocktail mix, followed by sonication at 4°C for 30 s. Lysates were centrifuged at 13,000 rpm for 10 min at 4°C, and supernatants were collected. Protein concentration was determined using the Pierce™ Bicinchoninic acid (BCA) Protein Assay Kit (Thermo Fisher Scientific, Rockford, IL, U.S.A.) following the manufacturer’s instructions.

### Assay optimization

Assay conditions, including buffer composition, pH, and choice of α-keto acid, were optimized individually for each substrate to reflect its known or experimentally determined enzymatic requirements. KYAT1 catalyzes diverse amino acid substrates, each with different reactivity profiles, stability, and preferred α-keto acid acceptors. For example, β-elimination reactions involving selenoamino acids (MSC and SeMet) were performed at alkaline pH (ammediol buffer, pH 9.0) to facilitate selenium bond cleavage, while transamination reactions were adjusted based on optimal conversion rates for each amino acid–keto acid pair. This substrate-specific approach allowed us to capture physiologically relevant activity patterns and minimize assay artifacts arising from non-ideal conditions.

#### Transamination assay for l-Phe

Transamination assay for l-Phe was performed according to a previously published protocol [42]. Briefly, 50 µl reaction containing 20 mM l-Phe, 5 mM KMB, 100 mM ammediol-HCl buffer (pH 9.0), and 200 ng of purified hKYAT1 or 20 µg of whole cell lysate protein. 0–100 mM l-Phe were used to determine Michaelis–Menten kinetics. Reactions were incubated at 37°C for 30 min and stopped by adding 150 µl of 1 M NaOH. Phenylpyruvate-enol (PPA-enol) formation was quantified by absorbance at 320 nm (*ε* = 16,000 M⁻¹cm⁻¹) using a PowerWave HT spectrophotometer (BioTek) and UV-transparent flat-bottom plates (Costar). Reactions without enzyme served as blanks.

#### Transamination assays for l-Leu, l-Ala, l-Cyss, l-Gln, dl-Met, Gly, Pro, SeMet, and MSC

The assays for l-Leu, l-Ala, l-Cyss, l-Gln, dl-Met, Gly, Pro, SeMet, and MSC were performed according to a previously published protocol [51]. Briefly, 50 µl reaction mixture containing 200 mM potassium phosphate EDTA buffer (pH 7.4), 5 mM Se-methylselenocysteine, and 0.6 mM PPA. The mixture was pre-incubated at 37°C for 5 min, followed by the addition of either 20 µg of whole-cell extract or 200 ng of purified hKYAT1 enzyme. 0–10 mM MSC or 0–15mM of SeMet were used to determine Michaelis–Menten kinetics. The reaction was incubated at 37°C for an additional 10 min and then terminated by adding 150 µl of 1 N NaOH. The consumption of phenylpyruvate was quantified by measuring the absorbance at 320 nm (*ε* = 16,000 M⁻¹cm⁻¹) using a PowerWave HT spectrophotometer (BioTek) in UV-transparent flat-bottom plates (Costar). Reactions without enzyme served as blanks.

#### Transamination of l-Trp, dl-Tyr, and l-His

Transamination of l-Trp, dl-Tyr, and l-His was measured following a modified version of the published protocol [52]. Briefly, 20 µg of whole-cell lysate or 200 ng of purified recombinant hKYAT1 was added to 100 µL of reaction mixture containing 3 mM amino acid, 5 mM KMB, 70 µM PLP, and 300 mM ammediol-HCl buffer (pH 9.6). 0–5 mM l-Trp were used to determine Michaelis–Menten kinetics. After incubation at 37°C for 30 min, the reaction was terminated by adding 7 µl of 50% trichloroacetic acid (TCA). The mixture was thereafter vortexed and centrifuged for 2 min at maximum speed. Then, 250 µl of 1 M arsenate–borate reagent (pH 6.0) was added to 50 µl of the reaction mixture, and the reaction mixture was vortexed and incubated for an additional 30 min at room temperature. Absorbance was acquired at 292 nm for l-His (imidazolepyruvate, ε = 11,300), 310 nm for dl-Tyr (p-hydroxyphenylpyruvate, ε = 10,700), and 330 nm for l-Trp (indolepyruvate, ε = 10,800) in a spectrophotometer (PowerWave HT, BioTek, U.S.A.). A mixture in which KMB was added immediately before adding 50% TCA was used as a blank.

#### Transamination of l-Kyn to l-Kyna

Trasamination of l-Kyn to l-Kyna with KBA as α-keto acid was performed using a 50 µl reaction mixture that contained 3 mM l-Kyn, 5 mM KBA, 200 µM PLP, and 300 mM ammediol-HCl buffer (pH 9.6), to which 40 µg of whole-cell lysate or 400 ng of recombinant hKYAT1 was added. 0–5 mM l-Kyn were used to determine Michaelis–Menten kinetics. The assay mixture was incubated at 37°C for 6 h, and the reaction was terminated by adding 3.5 µl of 50% TCA. The assay mixture was thereafter vortexed and centrifuged for 2 min at maximal speed. Then, 180 µl 500 mM phosphate buffer pH 7.5 was added to 20 µl of the reaction mixture, and absorbance was read immediately at 330 nm in a spectrophotometer (PowerWave HT, BioTek, U.S.A.). Under this condition, the molar extinction coefficient is 8850 M^−1^ cm^−1^ for l-Kyna [53]. A mixture in which KBA was added immediately before adding 50% TCA was used as a blank.

#### β-elimination activity for MSC and SeMet

β-elimination activity was measured for MSC and SeMet according to a previously published method [42]. Briefly, 100 µL reaction mixture contained 100 mM potassium phosphate buffer (pH 7.4), 5 mM MSC, 100 µM each of αKG and KMB, 10 µM PLP, 0.5 µg mammalian TrxR1, 400 µM NADPH, and either 200 ng of purified hKYAT1 or 20 µg of whole cell lysate protein. 0–10 mM MSC or 0–15mM of SeMet were used to determine Michaelis-Menten kinetics. After a 5 min pre-incubation at 37°C (excluding TrxR1 and NADPH), reactions were initiated by addition of TrxR1 and NADPH. NADPH consumption was continuously monitored at 340 nm every 30 sec using a PowerWave HT spectrophotometer (BioTek). A reaction without TrxR1 served as a blank. The molar extinction coefficient for NADPH was 6220 M⁻¹cm⁻¹. TrxR1 was added in excess to ensure saturation and allow accurate calculation of hKYAT1 kinetics.

### Immunoblotting

Western blot analysis was performed by loading 20 µg protein isolated from whole-cell lysates onto a 4–20% Mini-PROTEAN gel, followed by transfer to a PVDF membrane (Bio-Rad, U.S.A.). Membranes were blocked with 5% milk in TBST for two hours, then incubated with primary antibodies against KYAT1 (PA5-51313, 1:1000 in 5% BSA) (Sigma, Germany) overnight at 4°C. Vinculin (Millipore, U.S.A.) was used as a loading control. Membranes were washed and incubated for one hour with a secondary antibody, polyclonal swine anti-rabbit immunoglobulins/HRP (P0399) diluted 1:10,000 in 5% milk (DAKO, Denmark). Membranes were subsequently washed three times with TBST, and blot images were acquired using the Odyssey Fc Imaging System (LI-COR, U.S.A.). Protein levels were quantified using Odyssey Image software (LI-COR Biosciences, U.S.A.), by normalizing primary antibody signals to vinculin.

### Statistical analysis

Results are expressed as mean ± SD (*n* ≥ 3) and are displayed using box and whisker plots or violin plots. Statistical analyses were conducted using one-way ANOVA with a 95% confidence interval, followed by Dunnett’s multiple comparison test. Statistical significance was set at *P*<0.05. Data were analyzed using GraphPad Prism (version 10.1.2, GraphPad Software Inc., U.S.A.).

## Supplementary Material

Uncited online supplementary material 1

Online supplementary figure 1

Online supplementary figure 2

Online supplementary figure 3

Online supplementary table 1

## Data Availability

Data are available from the corresponding author upon request.

## Abbreviations

KP: kynurenine pathway
MSC: methylselenocysteine
PLP: pyridoxal 5´-phosphate
hKYAT1: human kynurenine aminotransferase 1
