# Supplementary material for: Mutational insights into human kynurenine aminotransferase 1: modulation of transamination and β-elimination activities across diverse substrates
Source: Biochem J. 2025 Aug 18;482(16):1163–80. doi: 10.1042/BCJ20253178 (PMC12493182; doi:10.1042/BCJ20253178)
Supplement: Uncited online supplementary material 1 [file bcj-482-16-BCJ20253178-s001.docx]

**Supplementary materials:**

**Supplementary Figure 1 . Michaelis-Menten kinetics of wild-type and five mutant recombinant hKYAT**. Transamination kinetics for L-Phe (n=6) **(A)**, L-Trp (n=3) (**B**), L-Kyn (n=3) (**C**), MSC (n=3) (**D**), and SeMet, (n=5) (**E**) and β-elimination kinetics for MSC (n=4) (**F**), and SeMet (n=9) (**G**)**.** *K*_m_ and *V*_max_ values were fitted using GraphPad Prism (v10.1.2). Each amino acid assay was performed in three to nine independent experiments.

**Supplementary Figure 2. Mutations in hKYAT1 do not affect transamination of certain amino acid substrates.** Transamination efficacy of thirteen different mutated variants of hKYAT1 was assessed for Tyr (3mM), Asp (3mM), Cyss (3mM) Pro (3mM), Ala (3mM), & Gly (3mM). (**A, C, E, G, I & K**) Cell lysates containing 20 µg of crude protein (n=5-7), and (**B, D, F, H, J & L**) wild-type and five recombinant mutant proteins (n=5-8), each at 200 ng, were used in the assays. (**A, C, E, G, I & K**) Bars are presented in the order: control (empty vector), wild-type hKYAT1, and followed by individual mutants. **(B, D, F, H, J & L)** Bars are presented in the order: wild-type hKYAT1, and followed by individual mutants. Statistical significance was determined using one-way ANOVA with a 95% confidence interval, followed by Dunnett’s multiple comparisons test (mean ± sd ns = not significant, * *p* < 0.05, ** *p* < 0.01, *** *p* < 0.001, and **** *p* < 0.0001 compared with wild-type hKYAT1 overexpressed lysate / wild-type hKYAT1 recombinant protein). Each amino acid assay was performed in five to eight independent experiments.

**Supplementary Figure 3. Expression levels of hKYAT1 mutant variants in HEPG2 cells.** (**A**) Western blot of hKYAT1 and mutant proteins from HEPG2 whole-cell lysates (20 µg) (n=5). (**B**) Quantification of hKYAT1 expression normalized to loading control vinculin. Statistical significance was determined using one-way ANOVA with a 95% confidence interval, followed by Dunnett’s multiple comparison test (mean ± sd ns = not significant, * *p* < 0.05, ** *p* < 0.01, *** *p* < 0.001, and **** *p* < 0.0001 compared with wild-type hKYAT1 overexpressed lysate, n=5 per group, represent five independent repeat).

**Supplementary Table 1:** Transamination and β-elimination activity of wild-type and mutant hKYAT1 with various amino acid substrates in crude cell extracts.

**Supplementary Table 2:** Protein sequences of hKYAT 1 wild-type and mutants.
