# Supplementary figures and images for: Mutational insights into human kynurenine aminotransferase 1: modulation of transamination and β-elimination activities across diverse substrates
Source: Biochem J. 2025 Aug 18;482(16):1163–80. doi: 10.1042/BCJ20253178 (PMC12493182; doi:10.1042/BCJ20253178)

Michaelis-Menten Kinetics

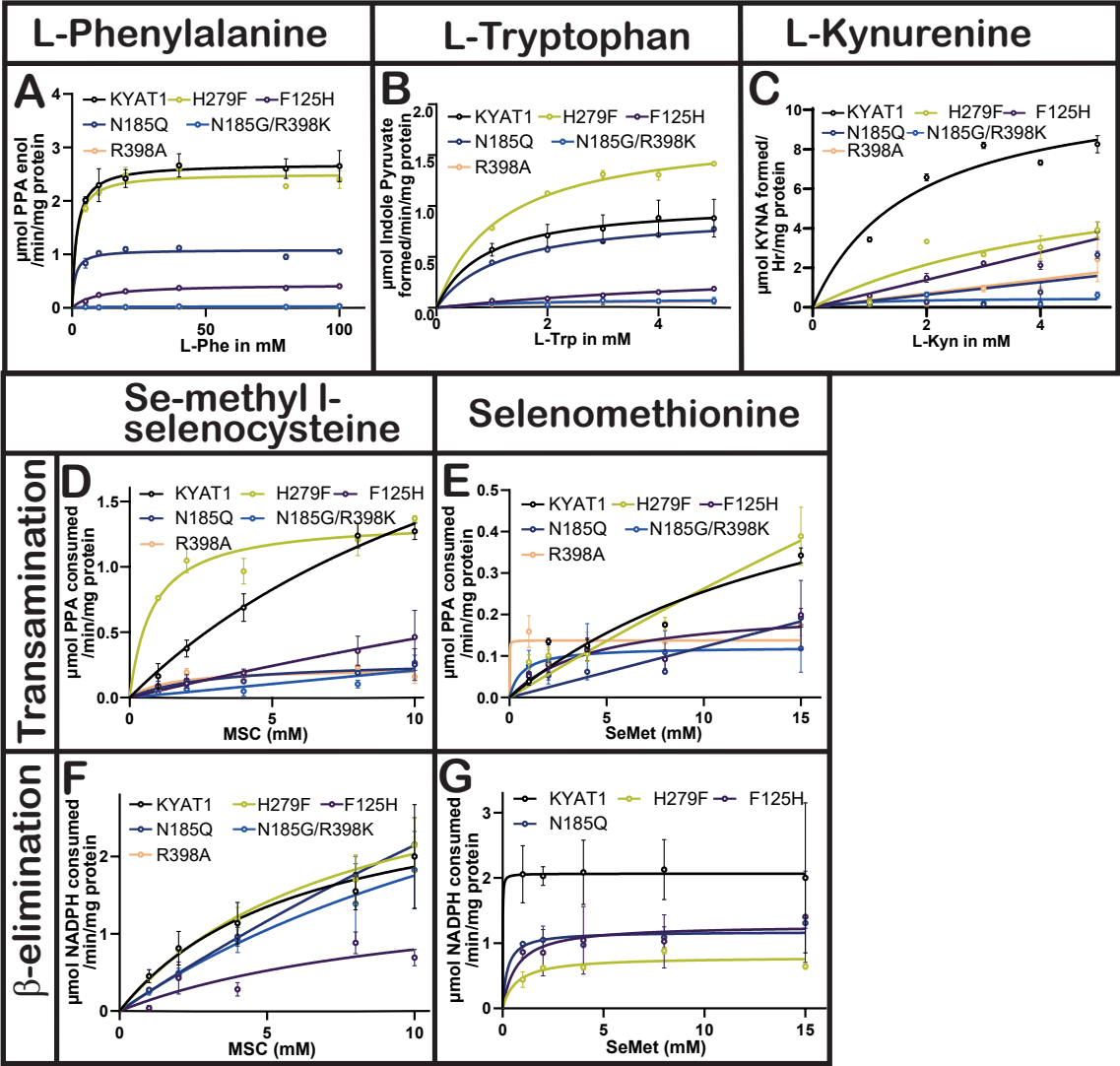

Supplement: Online supplementary figure 1 [file bcj-482-16-BCJ20253178-s001.pdf]

Supplementary figure 2

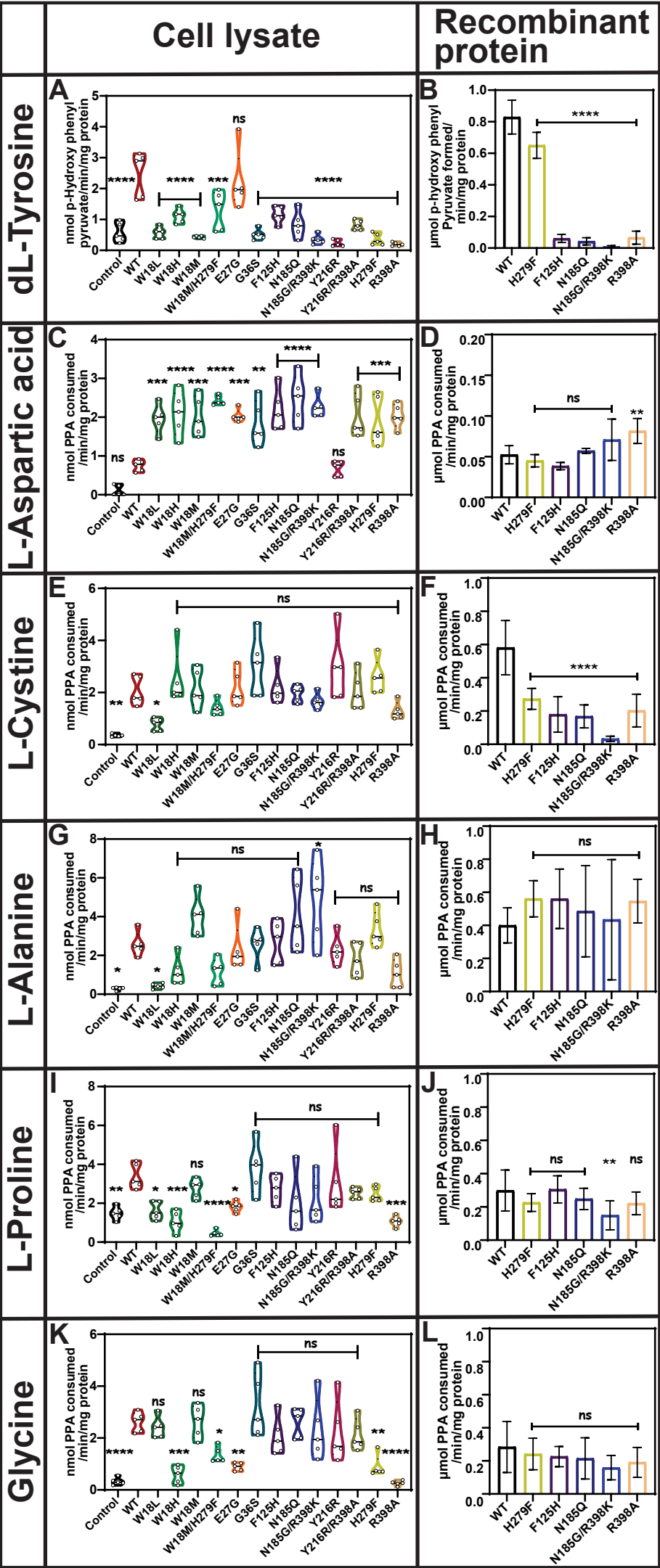

Supplement: Online supplementary figure 2 [file bcj-482-16-BCJ20253178-s002.pdf]

Supplementary  
figure 3

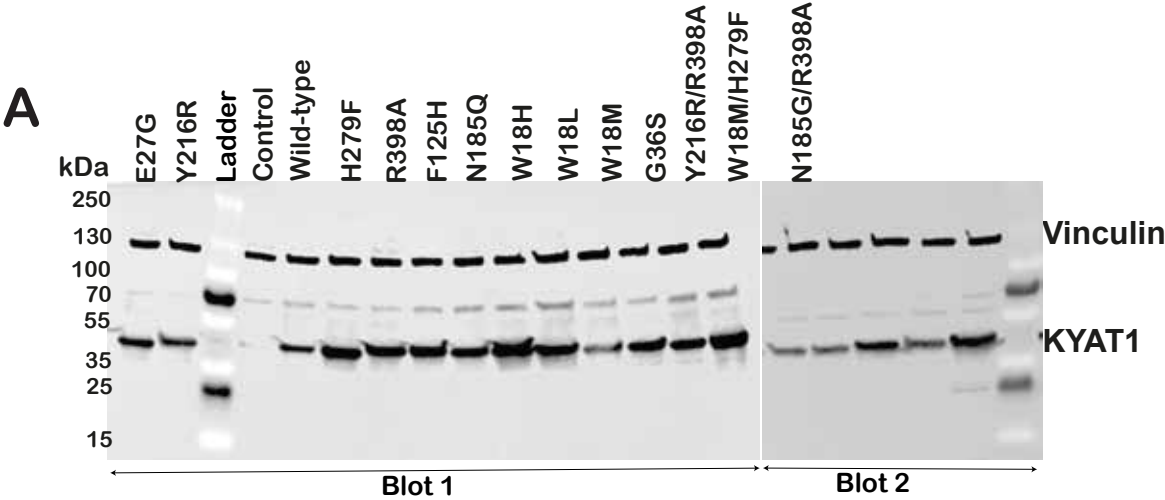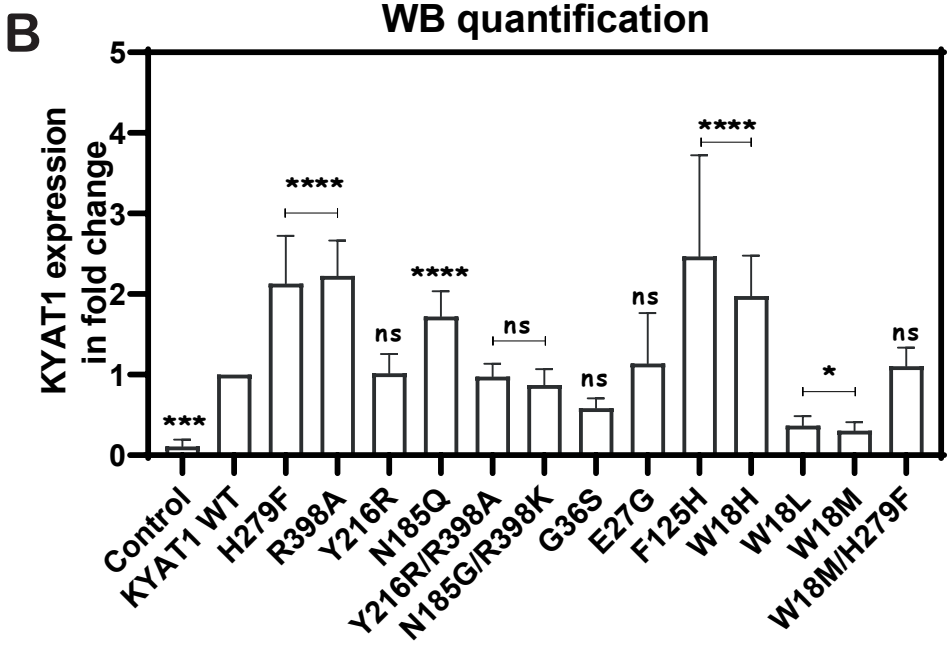

Supplement: Online supplementary figure 3 [file bcj-482-16-BCJ20253178-s003.pdf]
